# Efficacy of mebendazole in the spontaneous NZBxNZWF1 animal model of systemic lupus erythematosus

**DOI:** 10.1038/s41598-026-37930-z

**Published:** 2026-02-12

**Authors:** M. L. Eloranta, P. Nygren, R. Larsson, A. Loskog, N. Woodworth, M. Hultqvist, Richard Svensson, Ylva Gravenfors, L. Rönnblom, Mårten Fryknäs

**Affiliations:** 1 Department of Medical Sciences, Rheumatology, Uppsala University, Uppsala, Sweden; 2 Department of Immunology, Genetics and Pathology, Uppsala University, SE-75185 Uppsala, Sweden; 3 Department of Medical Sciences, Division of Cancer Pharmacology and Computational Medicine, Uppsala University, SE-75185 Uppsala, Sweden; 4 Redoxis AB, Medicon Village, Scheelevägen 2, SE-223 81 Lund, Sweden; 5 Department of Pharmacy, SciLifeLab Drug Discovery and Development, Uppsala University, SE-751 23 Uppsala, Sweden; 6 Science for Life Laboratory, Drug Discovery & Development Platform, Department of Biochemistry and Biophysics, Stockholm University, SE-171 21 Solna, Sweden

**Keywords:** Drug repositioning, Systemic lupus erythematosus, Mebendazole, Systemic lupus erythematosus, Drug development

## Abstract

Systemic lupus erythematosus (SLE) is a chronic autoimmune disease with a complex etiology involving both innate and adaptive immune dysregulation. Among several perturbed signaling pathways, decreased ERK activity in CD4⁺ T-cells has been linked to DNA hypomethylation and aberrant gene expression in SLE. Mebendazole (MBZ), an anti-helminthic drug with a well-established safety profile, has shown immunomodulatory effects in preclinical studies, including activation of the MEK/ERK pathway and inhibition of MAPK14 (p38), a known driver of inflammation. To evaluate the therapeutic potential of MBZ in SLE, we tested its efficacy in NZBxNZWF1 mice, a spontaneous and well-characterized SLE model. MBZ treatment resulted in reduced proteinuria, lower anti-dsDNA antibody levels, and diminished glomerular IgG deposition, both in preventive and therapeutic settings. Exploratory in vitro data suggest that MBZ may also influence ERK signaling in B cells, while the mechanistic basis of these effects remains to be clarified. Our findings demonstrate robust phenotypic improvements and support further investigation of MBZ as a repositioned candidate for SLE.

**Supplementary Information:**

The online version contains supplementary material available at 10.1038/s41598-026-37930-z.

## Introduction

Systemic lupus erythematosus (SLE) is a chronic autoimmune disease characterized by elevated levels of type I interferons (IFN) produced by plasmacytoid dendritic cells^1^ (pDCs), presence of autoantibodies targeting nucleic acid and associated nuclear proteins, and immune complex formation. These processes lead to inflammation and tissue damage in multiple organs^2^. SLE predominantly affects women of childbearing age and is known for its complex and multifactorial etiology.

Current treatment options for SLE include corticosteroids, antimalarials, and immunosuppressive drugs. More recently, biological agents have been introduced, such as anifrolumab and belimumab. Anifrolumab is a monoclonal antibody targeting the type I IFN receptor, thereby interfering with the expression of hundreds of IFN-regulated genes. Belimumab, a monoclonal antibody, inhibits the B-lymphocyte stimulator (BLyS), reducing the survival of autoreactive B cells and decreasing their differentiation into plasma cells. These treatments aim to downregulate the immunostimulatory properties of type I IFN and reduce the number of autoreactive B cells. However, a substantial proportion of patients with SLE show an incomplete response to these treatments^2^.

In SLE hypomethylated DNA is a common finding^3^. SLE can be triggered by hydralazine that is known to reduce DNA methylation via its inhibition of the extracellular signal-regulated kinase (ERK) signaling pathway^4^ demonstrating the important role of DNA hypomethylation in the disease course of SLE. Mebendazole (MBZ), a drug used to treat helminthic infections, has been shown to possess immunomodulatory properties, including activation of the mitogen-activated protein kinase (MEK)/ERK pathway in immune cells^5^. Defects in ERK signaling have been implicated in some autoimmune diseases including sarcoidosis^6^, but more evidently, in SLE^7^. Indeed, decreased ERK activity in CD4⁺ T-cells obtained from SLE patients can cause DNA hypomethylation but also aberrant gene expression associated with disease manifestation^7–10^. Moreover, a recent genome-wide association study has identified genetic variants near RASGRP1, a key upstream regulator of the ERK/MAPK signaling cascade, which are associated with SLE susceptibility and may contribute to impaired ERK activation in lymphocytes^11^. We have previously shown that MBZ can counteract the defective ERK signaling in CD4⁺ T-cells obtained from SLE patients^12^. In a recent study^13^, it was demonstrated how the MEK1 and MEK2 proteins, which activate ERK and are potential therapeutic targets for cancer and autoimmune diseases, regulate immune cell activation. The study showed that fine-tuning of the ERK/MAPK pathway is critical for regulating B- and T-cell activation as well as function, and that defective ERK activation results in typical SLE-like symptoms (i.e., anti-dsDNA antibodies and glomerulonephritis)^13^. MEK/ERK activation may also suppress type I interferon production by plasmacytoid dendritic cells (pDCs)^14^. In addition, MBZ has also been shown to directly bind and inhibit MAPK14 (p38), a well-known driver of autoimmune disease^15^. While these findings provide a rationale for testing MBZ in lupus models, direct mechanistic conclusions, particularly in vivo, remain speculative. Multiple pathways, including ERK activation and p38 inhibition, may contribute to its immunomodulatory effects.

Against this background, we aimed to investigate if MBZ treatment can reduce key clinical manifestations of SLE in a well-established in vivo model. The NZBxNZWF1 model is regarded as a gold standard for SLE as the mice spontaneously develop high levels of circulating anti-DNA autoantibodies which leads to formation of immune complexes causing lupus nephritis^16–18^. Two different time points for treatment were studied using the NZBxNZWF1 model; one preventive study, with early treatment onset (week 16–23), five weeks before disease manifestation, and one therapeutic study, with late onset treatment (week 28–36), at manifested disease. Treatment with MBZ was compared with methotrexate or anti-CD20 mIgG2a therapy. The general well-being of the animals, T and B cell populations in blood and spleen, proteinuria, anti-dsDNA antibodies, and immune complex deposits in the kidney were investigated.

## Results

In this study, we explored the therapeutic potential of mebendazole (MBZ) in a spontaneous NZBxNZWF1 mouse model for SLE. We assessed disease progression following both an early onset treatment and a late onset intervention with MBZ.

**Fig. 1:**
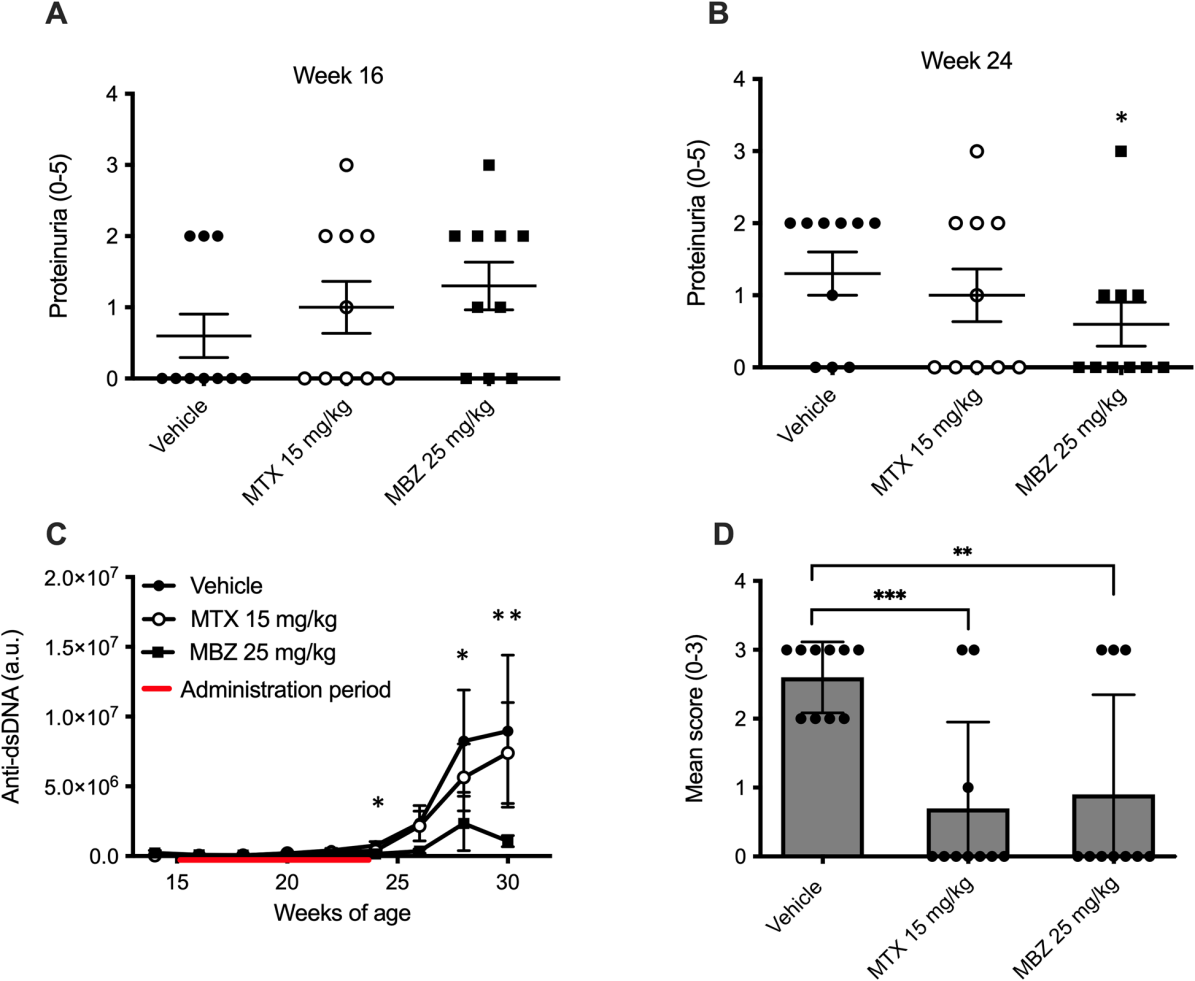
Effects of early-onset preventive treatment with mebendazole (MBZ) or methotrexate (MTX), administered between 16 and 23 weeks of age, on disease parameters in NZBxNZWF1 mice. (**A**, **B**) Proteinuria scores at (**A**) 16 weeks and (**B**) 24 weeks of age. For these panels, statistical comparisons were based on categorical outcomes, defined as the proportion of animals with proteinuria score ≥ 2, and analyzed using two-sided chi-square tests. (**C**) Serum anti–double-stranded DNA (anti-dsDNA) levels measured longitudinally throughout the study and presented as arbitrary units (a.u.). (**D**) Glomerular IgG deposition scores (0–3) assessed at study endpoint. Representative images corresponding to the scoring scale are shown in Supplementary Figure S3. For panels C and D, statistical comparisons were performed using two-sided non-parametric Mann–Whitney U tests, comparing treatment groups with vehicle. The treatment period is indicated by a red bar. Individual data points represent individual animals; horizontal lines indicate mean ± SEM. Asterisks denote statistical significance; *p < 0.05, **p < 0.01, ***p < 0.001.

In the early onset treatment part of the study, we observed a significant reduction of proteinuria in the 25 mg/kg MBZ treated animals at week 24 (*p* = 0.01) compared to animals treated with vehicle (Fig. 1A, B). Several animals treated with the high MBZ dose (50 mg/kg) showed a decline in general health, i.e. weight loss, kyphotic posture and fatigue. This group was excluded from the study and is not shown in comparative analyses, to maintain clarity in dose–response interpretation based on the analyzable 10 and 25 mg/kg groups (see Supplementary Table 1). All the remaining treatment groups exhibited a similar weight gain trajectory (Supplementary Fig. 1).

Furthermore, the serum anti-dsDNA levels observed during disease development were significantly lower in animals treated with MBZ compared to those treated with the vehicle (Fig. 1C). Fluorescence immunohistochemistry analysis for IgG antibody deposition in the glomeruli of the kidneys revealed markedly reduced IgG deposits in the glomeruli of mice treated with 25 mg/kg MBZ and methotrexate (Fig. 1D).

**Fig. 2:**
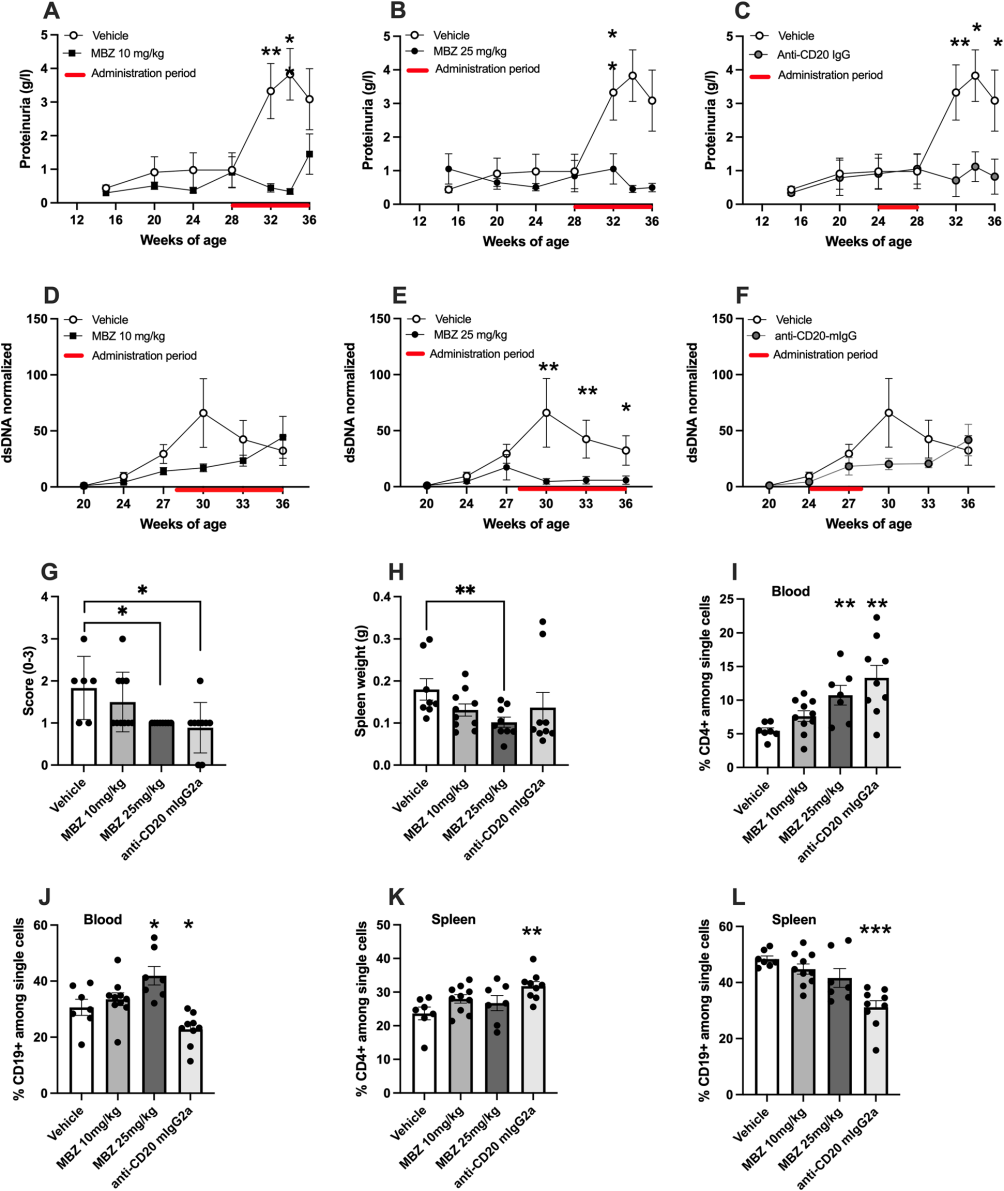
The effects of a late onset therapeutic treatment, between weeks 28 to 36 of age, with MBZ or anti-CD20 mIgG2a on SLE disease parameters and immune cell distributions in NZBxNZWF1 mice. (**A**–**C**) Proteinuria progression after treatment with (**A**) 10 mg/kg MBZ. (**B**) 25 mg/kg MBZ and (**C**) anti-CD20 mIgG2a, each compared to vehicle only. (**D**–**F**) Serum anti-dsDNA levels from week 20 to 36. (**D**) The effect of 10 mg/kg MBZ. (**E**) 25 mg/kg MBZ, and (**F**) 10 mg/kg anti-CD20 mIgG2a administered weekly from week 24 to 28 versus vehicle. (**G**) Glomerular IgG deposition scores from immunofluorescence analysis, see Supplementary Fig. S3 for representative images illustrating glomerular IgG scoring criteria. (**H**) Spleen weight comparison at study termination. (**I**–**L**) Distribution of immune cell populations at study termination. (**I** and **J**) show percentages of CD4⁺ and CD19⁺ cells in the peripheral blood, respectively. (**K** and **L**) show percentages of CD4⁺ and CD19⁺ cells in the spleen, respectively. Results are shown as mean ± SEM with asterisks indicating *p* values (*p < 0.05, **p < 0.01, ***p < 0.001). All endpoint analyses shown in panels (**G**–**L)** were performed at study termination (36 weeks of age). Sample size therefore varies between panels due to predefined humane endpoints and occasional insufficient biological material for specific endpoint analyses.

For the late onset treatment group, with focus on established disease, MBZ administration started at 28 weeks of age and we observed significantly lower levels of proteinuria between 32 and 34 weeks of age in the group of mice receiving 10 mg/kg MBZ (Fig. 2A) as well as at 34 weeks in the group treated with 25 mg/kg MBZ compared to the vehicle control (Fig. 2B). In addition, for comparative analysis, one subgroup was administered anti-CD20 mIgG2a from weeks 24 to 28 and showed significantly lower proteinuria at 32, 34, and 36 weeks of age in comparison with the vehicle (Fig. 2C), although a direct comparison of the used treatment regiments is complicated by the timing differences.

Notably, in the late onset treatment study with manifested disease, seven mice, three from the vehicle group, three from MBZ 25 mg/kg group and one animal from the anti-CD20 mIgG2a group were removed before the study was completed due to health issues. There was no difference in weight over time between groups in the late onset treatment study (Supplementary Fig. 2). No overt skin lesions or palpable lymphadenopathy were observed during routine monitoring.

Serum anti-dsDNA levels (Fig. 2D–F) during disease development were significantly lower in animals treated with 25 mg/kg MBZ (Fig. 2E) compared to the vehicle, whereas no such effect was detected with anti-CD20 mIgG2a treatment (Fig. 2F).

Significantly lower presence of IgG deposits was seen in the kidney glomeruli of mice treated with either 25 mg/kg MBZ or anti-CD20 mIgG2a compared to vehicle-treated animals at the end of the study (Fig. 2G). In addition, at the time point of study termination, the group administered 25 mg/kg MBZ was the only one to demonstrate significantly lower spleen weights compared to the vehicle control group in the late onset treatment study (Fig. 2H).

Furthermore, the percentages of CD4⁺ and CD19⁺ cells in peripheral blood and spleen were assessed using flow cytometry in the late onset treatment part of the study. In blood, both MBZ and anti-CD20 mIgG2a treatments significantly elevated the proportion of CD4⁺ cells (Fig. 2I). For CD19⁺ cells, anti-CD20 mIgG2a treatment resulted in a significant decrease, whereas MBZ (25 mg/kg) led to an increase in the proportion of CD19⁺ cells (Fig. 2J). In the spleen, only anti-CD20 mIgG2a treatment significantly raised the percentage of CD4⁺ cells (Fig. 2K) while reducing the proportion of CD19⁺ cells (Fig. 2L). Additionally, a non-significant trend towards an increase in CD4⁺ cells was observed with MBZ treatment (Fig. 2K).

**Fig. 3:**
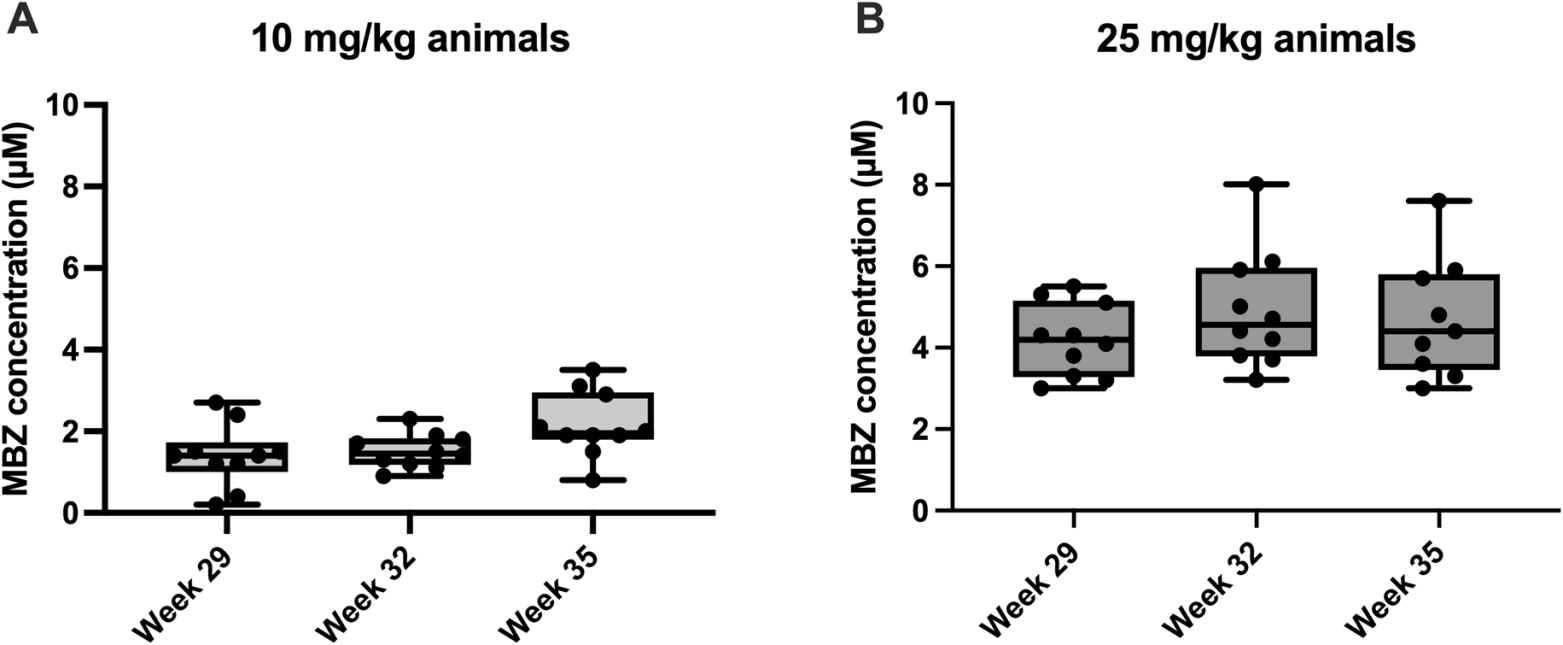
Evaluation of MBZ plasma levels in the late onset therapeutic study. (**A**) Box plot representation of plasma MBZ concentrations at weeks 29, 32, and 35 in animals dosed with 10 mg/kg. (**B**) MBZ concentrations for animals treated with 25 mg/kg MBZ. The boxes represent the range from the first to the third quartile with medians, whiskers extend to the minimum and maximum values, and individual data points are shown as dots. No statistical comparisons were performed for this figure.

In the late onset treatment study, plasma samples were obtained 4 h post-oral administration of MBZ, at weeks 29, 32, and 35 and revealed a dose-dependent exposure of MBZ. The concentration of MBZ in plasma was relatively stable across these measured timepoints, possibly with a tendency of accumulation by the end of the study period for the 10 mg/kg dose group (Fig. 3). This pharmacokinetic assessment offers insights into the systemic exposure of MBZ with the formulation used, allowing for potential correlations between drug levels, therapeutic efficacy, and safety profiles. These findings may also facilitate extrapolations of MBZ exposure in humans following anthelmintic treatment with this compound.

To explore potential mechanistic effects of MBZ beyond the in vivo findings, we performed an in vitro experiment using primary CD19⁺ B cells of murine splenic origin (not derived from the lupus model). Cells were exposed to MBZ (3 or 10 µM) for 1 h, and the phospho-ERK to total ERK ratio was quantified. A modest increase in the pERK/ERK ratio was observed at 3 µM MBZ, whereas the effect was less pronounced at 10 µM (Supplementary Fig. 4). Notably, the 3 µM concentration used in vitro is within the range of MBZ plasma concentrations measured in vivo, thereby supporting the physiological relevance of the experiment. While this experiment was limited in scope, the findings are consistent with previous reports and suggest that MBZ may modulate ERK signaling. These preliminary results provide exploratory context for the observed in vivo effects, but do not establish a mechanistic link.

## Discussion

In this study, we have demonstrated that MBZ exerts significant therapeutic activity in the NZBxNZWF1 mouse model of SLE, both as a preventative (i.e. early onset treatment) and therapeutic (i.e. late onset treatment) intervention. Consequently, we observed that treatment with MBZ significantly reduced anti-dsDNA antibody levels, proteinuria and immune complex depositions in the kidneys. In addition, we noted an increase in circulating B cells and CD4⁺ T cells after MBZ administration. Notably, the MBZ doses up to 25 mg/kg caused few side effects and did not affect the weight gain of the animals.

Due to the complexity, cost, and ethical considerations of the model, group sizes were limited to 8–10 animals. While this may affect statistical power, we observed consistent and statistically significant treatment effects across key disease parameters. The histopathological evaluation was limited to glomerular IgG deposition; future studies should include more detailed tissue scoring, including assessment of interstitial damage and perivascular infiltrates, immune cell phenotyping in renal tissue, as well as subclass-specific quantification of IgG isotypes to better characterize the humoral immune response.

The underlying mechanism(s) for the effect of MBZ in the present lupus model is unclear, but one possible interpretation is that MBZ may influence pathways associated with regulatory T-cell function in the NZBxNZWF1 mice. It has been documented that, Tregs are defective in lupus-prone NZW mice^19^ and that adoptive transfer of Tregs to NZBxNZWF1 mice prolong the survival of the animals after cyclophosphamide treatment^20^. Reduced Treg function is a prominent feature of many autoimmune diseases, including SLE^21^, which could be due to impaired MEK/ERK signaling as CD4⁺ T-cells in SLE patients exhibit impaired MEK/ERK signaling, leading to DNA hypomethylation and subsequent dysregulated gene expression^7–10^. Another possible contributing mechanism may involve p38 inhibition, which has been described for MBZ in other experimental systems^15^. However, no functional assays were performed to assess regulatory T cell activity, and our conclusions regarding immune modulation are therefore limited to descriptive phenotypic observations. This includes the potential immunological discrepancy that regulatory T cell activity and B cell expansion would not typically occur in parallel; however, such compartmentalized or indirect effects are not uncommon in complex autoimmune models. Another limitation is that no functional analysis of B cell activation or antibody production was performed, and the observed increase in CD19⁺ cells may not reflect enhanced humoral activity. An additional consideration is that mechanistic analyses were restricted to exploratory in vitro experiments using B cells from a normal mouse strain. Future studies should assess ERK signaling directly in B cells derived from NZBxNZWF1 animals and evaluate additional parameters such as in vivo ERK activity and levels of upstream regulators, including EGF. Likewise, more extensive histopathological assessment of renal lesions and measurement of total serum IgG concentrations could further refine the understanding of MBZ’s immunological and renal effects. These aspects were beyond the scope of the current work but represent important directions for future investigation.

MBZ has been observed to augment ERK signaling across various cell systems, including CD4⁺ T cells from SLE patients^11^, suggesting a potential pathway for its therapeutic effect. In the present study, we performed a complementary in vitro experiment using primary murine B cells, demonstrating a modest increase in ERK phosphorylation following MBZ exposure. In this in vitro assay assessing the effects of MBZ on ERK phosphorylation (Supplementary Fig. 4), the effect at 10 µM was weaker compared with 3 µM, which may reflect a non-linear (bell-shaped) dose–response, potential solubility or off-target issues, or short-term cellular stress under the assay conditions. Although these findings are preliminary and based on non-lupus-derived cells, they offer tentative mechanistic support for the notion that MBZ can modulate ERK signaling in immune cells. Furthermore, the ERK pathway is involved not only in Treg activation, but also in regulating B cell activation and survival^22^. Notably, sustained low-level ERK activation has been linked to the accumulation of autoreactive B cells, increased production of anti-dsDNA autoantibodies, and suppression of regulatory B cells^22^. Although additional targets such as type I IFN–producing cells may contribute to disease pathology in other lupus models, the NZBxNZWF1 strain does not display a strong type I IFN signature, and this axis was not investigated in the present study.

The need for new therapeutic targets in SLE treatment is pressing, since the approval of hydroxychloroquine in 1959, only a few, such as anifrolumab and belimumab, have been authorized, and their efficacy is not universal^2^. The scarcity of new drug approvals highlights the complexity of SLE and the challenges inherent in meeting the diverse medical needs of patients with SLE.

Although the underlying mechanisms remain to be clarified, our findings support further investigation into MBZ as a modulator of immune function in SLE, potentially involving CD4⁺ T cells and MEK/ERK signaling. Notably, we add exploratory in vitro data suggesting that MBZ may influence ERK signaling in primary murine B cells. Interestingly, the effects observed in this study were not replicated by another benzimidazole, fenbendazole^22^, as previously shown, suggesting that MBZ may exert unique immunomodulatory effects. In line with this, previous work has shown that MBZ, but not fenbendazole, inhibits p38, a kinase implicated in autoimmune and inflammatory responses^15^. This distinction may help explain the differing in vivo effects and highlights p38 inhibition as a potential mechanistic contributor to MBZ’s activity in SLE. In addition, affordability of MBZ and a favorable safety profile, even at high doses, make it a viable candidate for rapid clinical trial progression for an SLE indication. Leveraging the extensive existing pharmacokinetic and safety data can streamline the development process, significantly reducing the time and cost associated with clinical translation and potentially expediting the availability of a new treatment option for patients with SLE.

## Methods

### Animal husbandry

NZBxNZWF1 female mice (6–8 weeks of age) were acquired from Envigo, Europe. Mice (2–10 mice per cage) were housed in the animal facility (Medicon Village, Lund, Sweden) and kept at 12 h light/dark cycles. The mice were identified by chip insertion before study initiation.

### Health evaluation

Due to ethical reasons, mice with poor health status (e.g. dehydration, kyphotic posture, reduced mobility or other severe adverse effects) were euthanized. Euthanasia was performed by gradual exposure to increasing concentrations of carbon dioxide (CO_2_), without the use of anesthetic agents, in accordance with approved protocols and ethical permit. To ensure proper euthanasia, the animals were monitored for approximately 5 min, and circulation was checked by palpation.

### Animal weight, group assignments and treatments

In the early onset treatment study (preventive), the mice were weighed at 15 weeks of age whereas in the late onset treatment study (therapeutic), the mice were weighed at initiation of experiment (28 weeks of age). Due to mice arriving at different ages (4–6 weeks for early onset treatment and 6–8 weeks for late onset treatment groups, respectively) enrolment into the groups was done over three weeks.

#### Early onset treatment study

Forty female NZBxNZWF1 mice (6–8 weeks of age) were monitored for disease progression by weekly measurements of weight and levels of proteinuria as well as serum anti-dsDNA antibodies. At the age of 15 weeks the mice were randomized into four groups with 10 animals per group based on the weight and levels of anti-dsDNA antibodies.

The mice were given MBZ (25 or 50 mg/kg) or vehicle control p.o. daily for 5 consecutive days per week, during weeks 16–23 of age (a total of 8 weeks). For comparison methotrexate was administered i.p. three times weekly also during 16–23 weeks of age, in a fourth group. Urine sampling was performed every second week from start of treatment. Blood sampling was performed every 4th week until start of treatment and then every second week until end of experiment. Blood and kidneys were collected from all animals at termination, at the age of 32 weeks (half of the animals) or at the age of 30 weeks (half of the animals). Spleens were collected and weighed.

#### Late onset treatment study

Forty female NZBxNZWF1 mice (6–8 weeks of age) were monitored weekly as described above. Before the start of treatment, serum from mice of 20, 24 and 27 weeks of age were analyzed for levels of dsDNA to ensure disease progression. At age of 28 weeks, the mice were randomized into four groups, 10 animals per group, on account of weight and anti-dsDNA antibodies and commenced 9 weeks of treatment with MBZ, at either 25 mg/kg or 10 mg/kg p.o daily for 5 consecutive days per week or corresponding vehicle. For comparison, one group received anti-CD20 mIgG2a i.v. once per week from 24 to 28 weeks of age. Urine samples were analyzed for proteinuria first time at 15 weeks of age and then every four weeks from age 20 weeks until termination of the animals. Blood was collected every four weeks from 20 weeks of age until the end of the study. Blood, kidneys and spleens were collected from all animals at termination. The spleens were weighed and cells were prepared for flow cytometric analysis. The kidneys were processed for renal pathology by immunohistochemistry (IHC).

### Compound formulation and administration

MBZ was prepared fresh once weekly in 50:50 mix of PBS and sesame oil to final concentration corresponding to doses of 10, 25 and 50 mg/kg. anti-CD20 mIgG2a (3.8 mg/ml in sterile water) was administered i.v. corresponding to 10 mg/kg. Methotrexate was formulated in PBS and 0.1 ml was administered intraperitoneally (i.p.) to a final concentration of 15 mg/kg.

### Urine analysis

Combur 3 test strips (Roche) were used to determine proteinuria according to grading provided by vendor (negative = 0, 1 + = 0.3, 2 + = 1, 3 + = 5 g/l).

### Blood sampling

For welfare reasons, animals were lightly sedated with isoflurane (2%) prior to blood sampling, as NZBxNZWF1 mice are known to be particularly stress-sensitive. Blood was collected from the sublingual vein (under light anesthesia). For preparation of serum, blood was collected into gel containing micro tubes (Microvette 200, Z-gel) and centrifuged (10000×g, 5 min) within 60 min. For preparation of plasma, blood was collected in EDTA containing tubes (Microvette 200 K3, EDTA) and centrifuged (2000×g, 5 min) within 30 min. Serum and plasma were stored in aliquots at − 20 °C.

At termination blood was also collected in EDTA containing tubes (Microvette 200 K3, EDTA) and kept on ice before whole blood FACS analysis.

### Blood and spleen cell flow cytometry analysis

At termination of the animals in the late onset treatment study, blood and spleen cells were analyzed for B and T cell populations by flow cytometry. Single cell suspensions from spleens were prepared using 40 μm cell strainers and the piston of 1 ml syringes. Erythrocytes among splenocytes and in blood samples were lysed using 1x PharmLyse buffer and washed with PBS/1% BSA before staining with antibodies to CD4 and CD19 for 20 min at 4 °C. Cells were analyzed using the Attune Nxt flow cytometer (ThermoFisher Scientific).

### Anti-dsDNA ELISA

Poly-L-Lysine (20 µg/ml in TE buffer) coated 96-well microtiter plates were incubated with calf thymus DNA (20 µg/ml) over night at 4 °C, and blocked with 2% BSA in PBS for 1 h at room temperature (RT). After washing, standard (pooled serum) and samples were added and the plates were incubated at RT for 1.5 h. Subsequently, the plates were washed before horseradish peroxidase conjugated total IgG (250 ng/ml) was added and incubated for additional 1.5 h at RT. TMB was used as substrate and absorbance was measured at 650 nm.

### Tissue collection for assessment of renal pathology by fluorescent immunohistochemistry

At study termination, animals were euthanized by gradual exposure to increasing concentrations of carbon dioxide (CO_2_) prior to tissue collection. The left kidney from each animal was extracted and divided into two parts. One part was submerged in tissue embedding medium for cryosectioning, frozen in ice-cold isopentane and preserved at -80 °C. The other half was fixed in 4% formalin for 24 h, transferred to 70% ethanol and stored at 4 °C until processed. The spleens were collected and weight was determined before transferring them to ice cold PBS for flow cytometry analysis.

### Immunofluorescence staining of IgG

Cryosections (5 μm) prepared from kidneys embedded in OCT were mounted on Superfrost glass slides. Slides were dried at RT over night before storage at -80 °C. On the day of staining, slides were dried at RT before fixation in ice cold acetone for 2 min. Slides were dried at RT before washing in PBS for 5 min. The sections were blocked with 10% normal goat serum in PBS for 1 h in a wet chamber. The slides were then washed 2 × 5 min in PBS before incubation with FITC-conjugated goat anti-mouse IgG antibody diluted 1/100 in PBS with 5% normal goat serum for 1.5 h in a wet chamber in the dark. After washing the slides were mounted with Fluoroshield mounting medium and stored at 4 °C until microscopy. Images were acquired with an Olympus epifluorescence microscope and then evaluated blindly using a scale between 0 and 3 to define the IgG deposition in the kidney glomeruli, where 0 = not present, 1 = present, 2 = very present and 3 = clear glomerular accumulations. See Supplementary Fig. 3 for representative pictures of immunofluorescent staining of IgG deposition in kidney glomeruli.

### Bioanalysis of plasma samples by LC-MS/MS

Thawed plasma sample (25 µl) was mixed with 25 µl dH_2_O and precipitated in a 96-well plate with 150 µl ice-cold acetonitrile containing 100 nM warfarin as internal injection standard. The samples were centrifuged at 10 000 x g to produce a clear supernatant and immediately subjected for analysis. A linear standard curve was created in blank CD-1 mouse plasma by spiking from a 10 mM DMSO stock of MBZ and treated as above.

All quantitative analysis were performed using liquid chromatography coupled to triple-quad mass spectrometry (LC-MS/MS). Instrumentation used was a Waters TQs Micro coupled to a Waters Acquity UPLC. Mobile phases A: 99.9% ddH_2_O and 0.1% formic acid, B: 99.9% acetonitrile and 0.1% formic acid. The separation was achieved on a HSS T3 C18 2 × 50 mm column at 0.7 ml/min. Run time 2.5 min.

Gradient: 0 min/10% B − 0.5 min/10% B, 0.5 min/10% B − 1.5 min/60% B, 1.5 min/60% B − 1.8 min/95% B, 1.8 min/95% B − 2.2 min/95% B, 2.2 min/95% B − 2.4 min/10% B, 2.4 min/10% B − 2.5 min/10% B.

### In vitro stimulation of primary B cells and phospho-ERK analysis

Cryopreserved normal CD19⁺ B cells of murine splenic origin, isolated by positive selection for CD45R (cat. no. 3H2040, 3 H Biomedical, Sweden), were thawed and cultured according to the supplier’s instructions. Cells were plated at a density of 1.76 × 10^6^ cells per 500 µL in 24-well plates and treated for 1 h at 37 °C in RPMI 1640 medium supplemented with 10% fetal bovine serum, 2 mM L-glutamine, and 0.1% DMSO. Cells were exposed to vehicle (0.1% DMSO) or mebendazole (MBZ) at final concentrations of 3 µM or 10 µM, with or without co-treatment with EGF (100 ng/mL; BPS Bioscience, cat. no. 90201). Phospho- and total ERK1/2 levels were analyzed using the MILLIPLEX^®^ MAP Phospho/Total ERK 2-Plex Magnetic Bead Kit (cat. no. 48-619MAG, Merck) on a Luminex MAGPIX system (Bio-Rad). Protein concentration was measured using the Pierce™ BCA Protein Assay Kit (cat. no. 23227, Thermo Scientific), and samples were normalized accordingly. Results are expressed as phospho-ERK/total ERK ratio.

### Graphs and statistics

Graphs and statistical analyses were performed using GraphPad Prism 9 software (San Diego, CA, USA). Results are presented as mean values ± SEM, unless otherwise stated. Statistical comparisons were performed using two-sided non-parametric Mann–Whitney U tests for continuous or ordinal outcomes and two-sided chi-square tests for categorical outcomes, as specified in the corresponding figure legends. For figures presented descriptively, no statistical comparisons were performed. A p value < 0.05 was considered statistically significant, and significance levels were graphically indicated as follows: *p < 0.05, **p < 0.01, ***p < 0.001. Additional details on study design (randomization/blinding), sample size considerations, exclusions, and statistical analysis are provided in the Supplementary Information.

### Ethical permits

The local animal ethic committee Malmö/Lund, Sweden approved the study under the license 769/2020. Redoxis follow the EC policy as laid out in “Towards Responsible Animal Research” (EMBO R 4, 104–107), the Swedish Animal Welfare Act and SJVFS Guidelines 2019:9, L150, under the Swedish Board of Agriculture, Swedish animal welfare regulation and EU regulations (2010/63/EU Council Directive of 22 September 2010). All methods were performed in accordance with the relevant guidelines and regulations. Redoxis Internal guidelines were used for reporting, which are in concordance to the ARRIVE guidelines.

## Supplementary Information

Below is the link to the electronic supplementary material.

Supplementary Material 1

## Data Availability

Additional data to support the findings in this study are available as supplementary material or may be requested from the corresponding author.
