# Supplementary material for: Efficacy of mebendazole in the spontaneous NZBxNZWF1 animal model of systemic lupus erythematosus
Source: Sci Rep. 2026 Feb 12;16:6357. doi: 10.1038/s41598-026-37930-z (PMC12905218; doi:10.1038/s41598-026-37930-z)
Supplement: Supplementary file 1 — Supplementary Material 1 [file 41598_2026_37930_MOESM1_ESM.pdf]

Supplementary Information

Study design, randomization, and blinding

All animal experiments were conducted in accordance with approved ethical permits and institutional guidelines and are reported in line with the ARRIVE guidelines. Female NZBxNZWF1 mice were allocated to treatment groups using randomized group allocation based on body weight and serum anti–double-stranded DNA (anti-dsDNA) levels at baseline, to ensure balanced disease severity across groups at treatment initiation. Randomization was stratified by body weight to ensure an even distribution around the group mean, with all treatment groups represented within each cohort.

Investigators were not blinded to treatment allocation during dosing and routine monitoring. All downstream data analyses were performed blinded to treatment group, with animal identity as the only known parameter. However, evaluation of renal IgG deposition by immunofluorescence was performed in a blinded manner, with samples scored without knowledge of treatment group.

Sample size determination and exclusions

Group sizes were determined based on prior experience with the NZBxNZWF1 model, balancing statistical power with ethical considerations to minimize animal use. Group size considerations included the expected variability of key disease parameters and the duration of the treatment window. Typical group sizes were 8–10 animals per group.

Animals were excluded from analysis only if predefined humane endpoints were reached due to deteriorating health, in accordance with ethical guidelines. Animals euthanised before the planned study endpoint were included in analyses up to the time point of removal. Exclusions were not based on experimental outcomes and occurred across treatment groups, as described in the Results section. No statistical imputation was performed for missing data; excluded animals were omitted from subsequent analyses.

Outcome measures

Primary outcome measures were proteinuria, serum anti-dsDNA antibody levels, and glomerular IgG deposition. Secondary outcome measures included spleen weight and distributions of $CD4^+$ and $CD19^+$ immune cell populations in blood and spleen. Exploratory

outcomes included plasma mebendazole exposure and in vitro ERK phosphorylation in primary murine B cells.

Statistical considerations

Statistical analyses were performed as specified in the main Methods section and in the corresponding figure legends. Continuous or ordinal outcomes were analyzed using two-sided non-parametric Mann–Whitney U tests, while categorical outcomes (e.g., the proportion of animals with proteinuria score ≥2) were analyzed using two-sided chi-square tests, reflecting the scale and distribution of the data.

Figures intended to be descriptive or exploratory were not subjected to statistical testing, as explicitly stated in the figure legends. A p value <0.05 was considered statistically significant.

Data handling and reporting

All data are presented as mean ± SEM unless otherwise stated. Individual data points represent individual animals. No outlier exclusion was performed beyond the predefined humane endpoints described above.

Supplementary Figure 1. Body weight progression from weeks 10 to 30 in the early-onset treatment study (vehicle, MTX 15 mg/kg, MBZ 25 mg/kg). Data are shown as mean ± SEM. Weight gain trajectories were consistent across all groups. No statistical comparisons were performed.

Supplementary Figure 2. Body weight progression from weeks 15 to 35 in the late-onset therapeutic study (vehicle, MBZ 10 mg/kg, MBZ 25 mg/kg, anti-CD20 IgG 10 mg/kg). Data are shown as mean ± SEM. Weight gain trajectories were consistent across all groups. No statistical comparisons were performed.

Supplementary Figure 3. Representative images of immunofluorescent IgG staining in kidney glomeruli, illustrating the glomerular IgG deposition scoring criteria (0–3), where 0 = not present, 1 = present, 2 = very present, and 3 = clear glomerular accumulations. Scale bar: 50 μm.

Supplementary Figure 4. MBZ exposure increases ERK phosphorylation in primary murine B cells. Cryopreserved $CD19^+$ B cells of murine splenic origin were cultured in vitro and treated with MBZ (3 or 10 μM) for 1 hour. Epidermal growth factor (EGF, 100 ng/mL, 1 hour) served as a positive control. Cells were lysed and analyzed using a Luminex-based multiplex assay to quantify phosphorylated and total ERK, and the pERK/ERK ratio was calculated for each condition. Data represent mean ± SD of duplicate wells. No statistical testing was performed (n = 1 independent experiment); results are exploratory and should be interpreted accordingly.

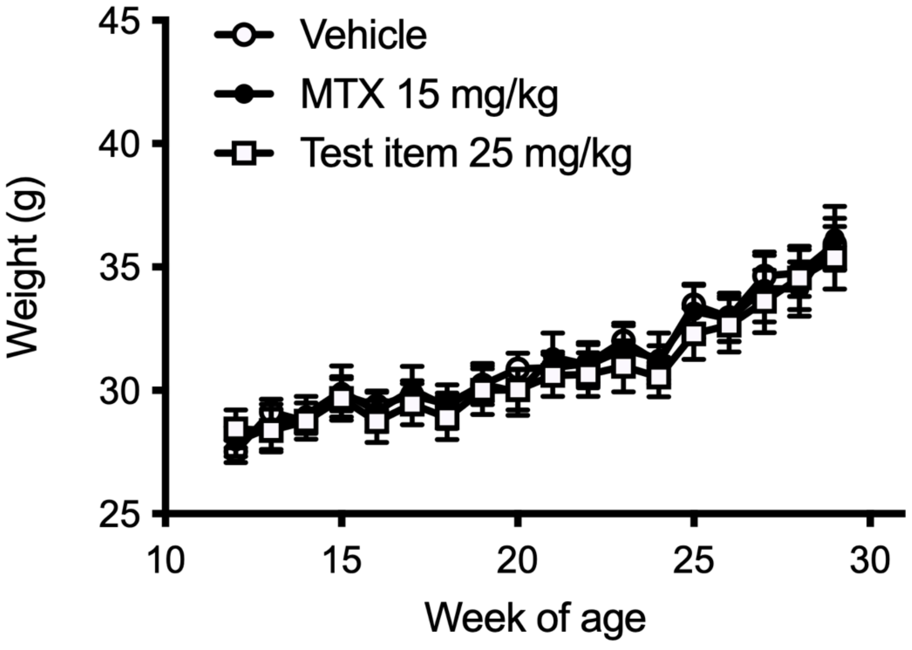

Supplementary Figure 1.

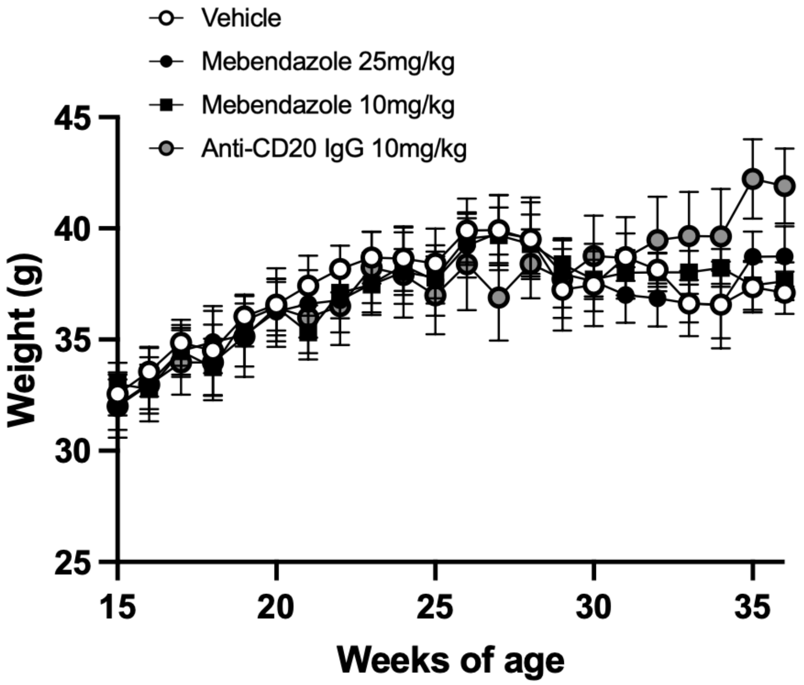

Supplementary Figure 2.

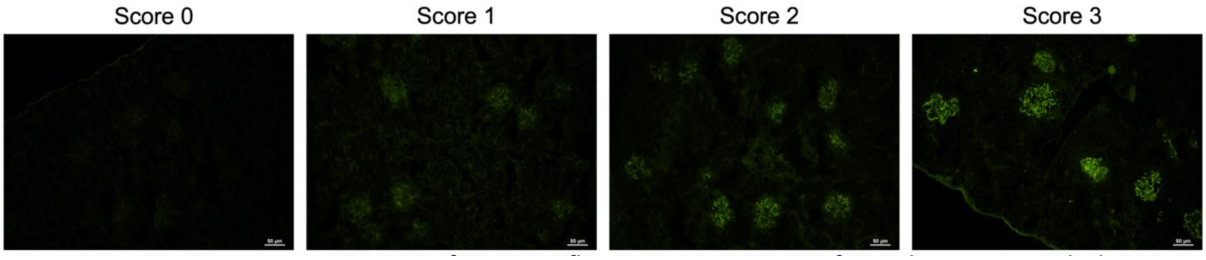

Supplementary Figure 3.

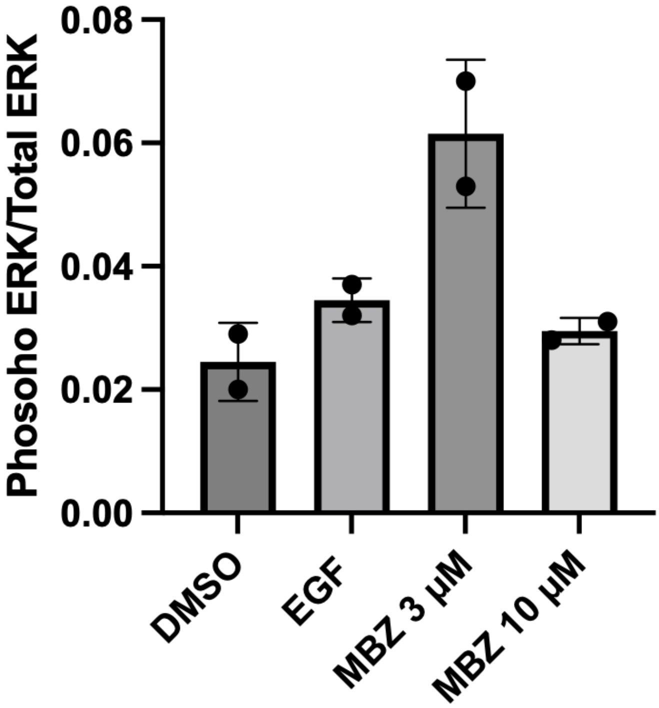

Supplementary Figure 4.

**Supplementary Table 1. Clinical observations and termination time points in the 50 mg/kg MBZ treatment group**			
**Animal ID**	**Treatment**	**Comment**	**Week**
**B5**	50 mg/kg	Kyphotic posture, weakness	22
**B6**	50 mg/kg	Found dead	18
**C7**	50 mg/kg	Kyphotic posture, weakness, impaired circulation	19
**D3**	50 mg/kg	Kyphotic posture, weakness	22
**D9**	50 mg/kg	Found dead	22

Five animals treated with 50 mg/kg mebendazole (MBZ) were prematurely terminated due to declining health. Clinical signs included kyphotic posture, weakness, impaired circulation, or sudden death. The table below lists the animal IDs, observed clinical signs, and the study week at which the animal was terminated.
